# Prehabilitation and Postoperative Outcomes in Major Surgery: A Retrospective Cohort Study Using MarketScan Claims Data

**DOI:** 10.1002/wjs.70437

**Published:** 2026-06-09

**Authors:** Meher Angez, Zayed Rashid, Odysseas P. Chatzipanagiotou, Areesh Mevawalla, Elemosho Abdulaziz, Qaidar Alizai, Timothy M. Pawlik

**Affiliations:** ^1^ Department of Surgery The Ohio State University Wexner Medical Center and James Comprehensive Cancer Center Columbus Ohio USA; ^2^ Department of Internal Medicine Conemaugh Memorial Medical Center Johnstown Pennsylvania USA

## Abstract

**Background:**

Prehabilitation, including preoperative exercise, nutrition optimization, behavioral support, and smoking cessation services, may improve physiological reserve in patients undergoing surgery; however, large administrative and claims‐based real‐world evaluations across diverse procedure types have remained limited.

**Methods:**

This retrospective cohort study used IBM MarketScan claims (2010–2020) to identify adults undergoing coronary artery bypass grafting (CABG), abdominal aortic aneurysm (AAA) repair, pneumonectomy, pancreatectomy, and colectomy. Prehabilitation was defined as claims for physical therapy, nutrition counseling, psychological/behavioral counseling, and smoking cessation services within 30–90 days preoperatively. Multivariable logistic regression assessed outcomes, including any major postoperative complication, readmission, and costs, adjusting for baseline patient and hospital factors.

**Results:**

Among 136,674 patients, 6.6% (*n* = 9077) received prehabilitation. The median age was 56 (49–60), and 42.0% (*n* = 57,035) were female. Patients who received prehabilitation more commonly underwent pneumonectomy (15.8% vs. 11.3%) or AAA repair (4.7% vs. 3.2%) and received care at urban/metropolitan hospitals (86.9% vs. 84.7%) compared with individuals who did not undergo prehabilitation (all *p* < 0.001). Prehabilitation was associated with lower odds of any index complication (aOR 0.94, 95% CI 0.90–0.99) and lower risk of myocardial infarction (MI) at index hospitalization (aOR 0.78, 95% CI 0.71–0.85), 30 days (aOR 0.79, 95% CI 0.72–0.86), and 90 days (aOR 0.81, 95% CI 0.74–0.89). Moreover, patients who received prehabilitation experienced higher costs, both preoperatively (*β* 4227, 95% CI 3834–4621) and postoperatively (*β* 4020, 95% CI 2866–5173). In sub‐analyses, the association between prehabilitation and lower odds of MI was maintained among CABG patients (aOR 0.83, 95% CI 0.76–0.90).

**Conclusions:**

Prehabilitation was associated with lower postoperative cardiac events but higher perioperative costs; the most favorable associations were observed among CABG patients, although these subgroup findings should be interpreted cautiously.

## Graphical abstract

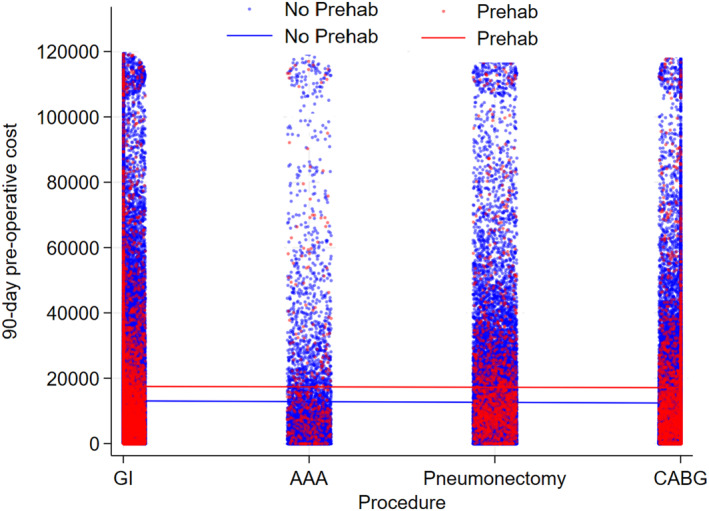

## 1 Introduction

Despite advances in anesthetic techniques and enhanced recovery pathways, approximately one in four patients undergoing a major surgical procedure experience postoperative complications [1, 2]. Patients with frailty, malnutrition, anemia, or sarcopenia are particularly vulnerable due to diminished physiologic reserves, placing them at higher risk for postoperative infections, impaired healing, and prolonged recovery [3]. Prehabilitation is a personalized, multimodal, needs‐driven strategy designed to improve physiological and psychological reserves before surgery. Prehabilitation aims to prepare patients for common perioperative stressors such as anesthesia, surgical instrumentation, neoadjuvant therapy, fluid shifts, and oxidative stress [1, 2, 4]. The intervention is typically based on three domains—physical conditioning, nutritional optimization, and psychological support—and typically includes components such as aerobic and resistance training, inspiratory muscle training, protein supplementation, smoking cessation, and anxiety reduction strategies [5, 6].

Clinical evidence suggests that preoperative optimization can improve outcomes in abdominal and other major surgeries, with prehabilitation programs associated with fewer postoperative complications and shorter hospital stays [6, 7, 8]. For instance, Boden et al. reported that structured preoperative exercise reduced pulmonary complications and inpatient length of stay (LOS) [9]. Similarly, Martin et al. noted that a multimodal prehabilitation program was linked to fewer complications, shorter LOS, and lower hospitalization costs than standard of care [10]. Physical prehabilitation—particularly aerobic and inspiratory muscle training—has also been linked to lower overall morbidity and pneumonia risk, while shortening LOS by approximately 1–2 days [11, 12].

To date, much of the evidence supporting prehabilitation has come from trials, meta‐analyses, and single‐center studies, whereas large administrative or claims‐based evaluations across multiple high‐risk operations have remained limited [13, 14]. To address this gap, we conducted a large‐scale, real‐world analysis using national administrative claims data to evaluate the impact of prehabilitation on perioperative outcomes and costs among patients undergoing a major surgical procedure. The objective of the current study was to assess the effectiveness of prehabilitation across diverse clinical settings and surgical procedure types with the goal of informing clinical practice and policy decisions regarding the value of preoperative optimization.

## 2 Methods

### 2.1 Data Source and Study Population

Adults (≥ 18 years) who underwent a major surgical procedure between January 1, 2010 and December 31, 2020 were identified from the IBM MarketScan Commercial Claims and Encounters database. This large de‐identified repository includes inpatient, outpatient, and pharmacy claims from employer‐sponsored insurance plans across the United States [15]. The database longitudinally captured diagnoses, procedures, healthcare utilization, and payments for individual enrollees and their dependents. The following major surgical procedures were selected a priori based on clinical risk in and frequent ascertainment about claims: coronary artery bypass grafting (CABG), open abdominal aortic aneurysm (AAA) repair, pneumonectomy, pancreatectomy, and colectomy. Procedures were identified using ICD‐10‐PCS root code prefixes across all available procedure fields (Supporting Information S1: Table S1) [16, 17]. These procedures were selected to represent high‐risk inpatient operations across cardiac, vascular, thoracic, pancreatic, and colorectal surgery that are associated with substantial perioperative morbidity, have a clearly identifiable index admission in claims, and occurred with sufficient frequency to permit stable risk‐adjusted analyses. Other major operations were not included because of lower case volume, greater coding heterogeneity, or overlapping with broader procedure groupings analyzed in the current study. Patients were included if they were continuously enrolled for at least 6 months before and 3 months after surgery to ensure complete capture of exposure and outcome data. No prior sample size calculation was performed because this retrospective, claims‐based cohort study included all eligible adults (≥ 18 years) undergoing major surgery in the IBM MarketScan Commercial Claims and Encounters database from January 1, 2010 to December 31, 2020. Given the large underlying population captured in MarketScan, the analytic cohort provided substantial statistical power and precise estimates, allowing detection of modest differences in postoperative outcomes. The study used de‐identified data; the need for informed consent was deemed exempt by the Ohio State University Institutional Review Board. Reporting followed STROBE guidelines.

### 2.2 Primary Exposures and Outcomes of Interest

Prehabilitation service claims included (1) exercise/physical therapy (e.g., CPT 97110, 97112, and 97116), (2) nutrition counseling/dietitian services (CPT 97802, 97803, and G0270), (3) psychological/behavioral counseling (CPT 96152–96154, 99401–99404), and/or (4) smoking cessation counseling (CPT 99406–99407) [18]. Prehabilitation was defined as receipt of ≥ 1 such service within 90 days before the index surgery and categorized as none, bimodal (2 modalities), or multimodal (≥ 3 modalities) to evaluate a potential dose–response relationship [18]. These claims‐based services were used as proxies for prehabilitation; however, the database could not confirm whether each service was delivered specifically as part of a coordinated preoperative prehabilitation program.

Primary outcomes of interest were (1) a composite end point encompassing any major postoperative complication, and (2) individual complications, including cardiac (myocardial infarction [MI]), respiratory (pneumonia and respiratory failure), thromboembolic (deep vein thrombosis and pulmonary embolism), and renal (i.e., acute kidney injury) complications [19, 20]. Complications were identified using diagnosis and procedure codes during index hospitalization, as well as within 30 and 90 days postoperatively (Supporting Information S1: Table S2). Secondary outcomes included in‐hospital mortality, inpatient LOS, 30‐ and 90‐day all‐cause readmissions (nonelective), and costs, categorized as (a) 90‐day preoperative and (b) 90‐day postoperative medical costs [18, 21]. Charges were converted to costs using paid amounts available in MarketScan and inflation‐adjusted to 2020 U.S. dollars using the Personal Health Care Index [22].

### 2.3 Covariates

Covariates included patient age (i.e., 18–34, 35–49, 50–64, and ≥ 65 years), sex, U.S. region (i.e., Northeast, Midwest, South, and West), insurance type (i.e., PPO, HMO, and high‐deductible), year of surgery, surgery type (i.e., AAA repair, CABG, lung resection, pancreatectomy, and colectomy), and admission type (i.e., elective and nonelective). The comorbidity burden was summarized using the Charlson comorbidity index (CCI) (i.e., 0, 1–2, and ≥ 3) based on ICD codes recorded within 6 months before surgery. Specific comorbidities relevant to postoperative risk (e.g., diabetes, chronic pulmonary disease, and heart failure) were also identified [23]. Hospital characteristics, including teaching status and urban or rural location, were obtained from MarketScan provider files.

### 2.4 Statistical Analysis

Continuous variables were summarized as median values with interquartile ranges (IQRs) and categorical variables as counts with percentages (%). The Mann–Whitney *U*‐test was used to compare continuous variables, and the Fischer exact test as used for categorical variables. Multivariable logistic regression was used to assess risk‐adjusted associations between prehabilitation and binary outcomes (any complication, specific complications, readmission, and mortality). Results were presented as adjusted odds ratios (aORs) with 95% confidence intervals (CIs). Generalized linear models with a gamma distribution and log link—due to right skewness in the data distribution—were used to model LOS and cost outcomes. Estimates were reported as adjusted differences or % differences with 95% CIs. All models were adjusted for age, sex, CCI, insurance type, region, surgery year, surgical category, admission type, hospital teaching status, and urbanicity. Robust standard errors were used, and sensitivity analyses included stratification by the number of prehabilitation modalities (i.e., 0, 1, 2, and > 2), as well as surgical procedure types. All tests were two‐sided, and *p* < 0.05 was deemed statistically significant. Analyses were performed using SAS 9.4 (SAS Institute, Cary, NC).

## 3 Results

### 3.1 Baseline Characteristics

Among 136,674 patients who underwent a major surgical procedure (CABG: *n* = 39,503, 28.9%; pneumonectomy: *n* = 15,859, 11.6%; AAA repair *n* = 4454, 3.3%; pancreatectomy *n* = 4881, 3.6%; and colectomy: *n* = 71,977, 52.7%), the median age was 56 years (IQR 49–60), most individuals were male (*n* = 79,639, 58.3%), and 60.6% (*n* = 82,759) of patients had CCI > 3. Most individuals were covered by employer‐sponsored plans (*n* = 92,734, 67.8%), with PPOs being most common (*n* = 78,595, 57.5%). Hospitals were predominantly located in the South (*n* = 64,828, 47.4%) and in metropolitan areas (*n* = 115,974, 84.7%) (Table 1).

**TABLE 1:** Baseline characteristics of patients undergoing major surgery, by prehabilitation status.

Patient characteristics	Overall	No prehabilitation	Prehabilitation	*p*‐value
	*N* = 136,674	*N* = 127,597 (93.4)	*N* = 9077 (6.6)	
Age, years	56 [IQR: 49–60]	56 [IQR: 49–60]	56 [IQR: 50–61]	< 0.001
18–34	7328 (5.4)	6972 (5.5)	365 (3.9)	
35–49	26,922 (19.7)	25,225 (19.8)	1697 (18.7)	
50–64	102,289 (74.8)	95,275 (74.7)	7014 (77.3)	
≥ 65	135 (0.10)	125 (0.10)	10 (0.11)	
Sex				< 0.001
Male	79,639 (58.3)	74,874 (58.7)	4765 (52.5)	
Female	57,035 (41.7)	52,723 (41.3)	4312 (47.5)	
Charlson comorbidity index				0.79
0	239 (0.2)	224 (0.2)	15 (0.2)	
1–2	53,676 (39.3)	50,140 (39.3)	3536 (39.0)	
> 3	82,759 (60.6)	77,233 (60.5)	5526 (60.9)	
Type of procedure				< 0.001
CABG	39,503 (28.9)	37,045 (29.0)	2458 (27.1)	
Pneumonectomy	15,859 (11.6)	14,429 (11.3)	1430 (15.8)	
AAA	4454 (3.3)	4028 (3.2)	426 (4.7)	
Pancreatectomy	4881 (3.6)	4552 (3.57)	329 (3.6)	
Colectomy	71,977 (52.7)	67,543 (52.9)	4434 (48.8)	
Insurance coverage				< 0.001
Comprehensive	5557 (4.1)	5176 (4.1)	381 (4.3)	
EPO	1609 (1.2)	1507 (1.2)	102 (1.1)	
HMO	15,037 (11.0)	14,181 (11.3)	856 (9.6)	
POS	9626 (7.0)	8963 (7.1)	663 (7.4)	
PPO	78,595 (57.5)	73,359 (58.4)	5236 (58.4)	
POS (capitated)	1209 (0.9)	1151 (0.9)	58 (0.6)	
CDHP	14,145 (10.4)	13,141 (10.4)	1004 (11.2)	
HDHP	1983 (1.45)	8247 (6.6)	658 (7.4)	
Health plan indicator				< 0.001
Employer sourced	92,734 (67.8)	86,310 (67.6)	6424 (70.8)	
Health plan sourced	43,940 (32.2)	41,287 (32.4)	2653 (29.2)	
Hospital region				< 0.001
Northeast	23,153 (16.9)	21,422 (16.8)	1731 (19.1)	
North central	30,014 (22.0)	28,042 (22.0)	1972 (21.7)	
South	64,828 (47.4)	60,793 (47.6)	4035 (44.4)	
West	17,274 (12.6)	16,022 (12.6)	1252 (13.8)	
Unknown	1405 (1.0)	1318 (1.0)	87 (1.0)	
Hospital location				< 0.001
Urban/metropolitan	115,974 (84.8)	108,088 (84.7)	7886 (86.9)	
Rural/nonmetropolitan	20,700 (15.2)	19,509 (15.3)	1191 (13.1)	
Modality count				
2 modalities	—	—	5472 (60.3%)	
≥ 3 modalities	—	—	3605 (39.7%)	

*Note:* Values are presented as counts (%) unless otherwise indicated.

Abbreviations: AAA, abdominal aortic aneurysm; CABG, coronary artery bypass graft; CCI, Charlson comorbidity index; CDHP, consumer‐driven health plan; EPO, exclusive provider organization; HDHP, high‐deductible health plan; HMO, health maintenance organization; LOS, length of stay; POS, point of service; POS (capitated), point of service (capitated); PPO, preferred provider organization; SD, standard deviation.

Overall, 6.6% (*n* = 9077) of patients received prehabilitation prior to major surgery among whom 60.3% (*n* = 5472) participated in two services, and 39.7% (*n* = 3605) in three or more. Prehabilitation recipients were more often female (47.5%, *n* = 4312 vs. 41.3%, *n* = 52,723) and more likely to have a planned pneumonectomy (15.8%, *n* = 1430 vs. 11.3%, *n* = 14,429) or AAA repair (4.7%, *n* = 426 vs. 3.2%, *n* = 4028) than individuals who did not receive prehabilitation (both *p* < 0.001). Moreover, prehabilitation was more common among patients with employer‐sponsored insurance (70.8%, *n* = 6425 vs. 67.6%, *n* = 86,310) and individuals treated at urban/metropolitan hospitals (86.9%, *n* = 7886 vs. 84.7%, *n* = 108,088) (both *p* < 0.001). In contrast, prehabilitation use was less frequent among patients enrolled in HMOs (9.6%, *n* = 856 vs. 11.3%, *n* = 14,181) or capitated POS plans (0.6%, *n* = 58 vs. 0.9%, *n* = 1151) (*p* < 0.001). No difference in CCI was observed between the two groups (*p* = 0.79).

### 3.2 Unadjusted Outcomes Across Groups

During index hospitalization, patients who did versus did not undergo prehabilitation experienced a slightly lower overall incidence of in‐hospital complications (25.3% vs. 26.9%; *p* = 0.001), driven primarily by a lower incidence of MI (8.1% vs. 10.1%; *p* < 0.001); there were no observed differences relative to respiratory, thrombotic, or renal complications (all *p* > 0.05) (Table 2). Over the 30‐day postoperative window that included post‐discharge, the overall incidence of complications remained similar among patients who did and did not receive prehabilitation services (31.4% vs. 32.4%; *p* = 0.057) except for MI, which was less frequent among individuals who had prehabilitation (8.7% vs. 10.7%; *p* < 0.001). A similar pattern of overall comparable morbidity was observed at 90‐days (*p* = 0.285), yet a lower MI incident among patients who underwent prehabilitation (9.3% vs. 11.2%; *p* < 0.001) was noted.

**TABLE 2:** Postoperative outcomes among patients who received prehabilitation.

Outcomes	No prehabilitation	Prehabilitation	*p*‐value
	*N* = 127,597 (93.4)	*N* = 9077 (6.6)	
Index date complication (any)			
Any major complication	34,308 (26.9)	2298 (25.3)	0.001
MI	12,912 (10.1)	735 (8.1)	< 0.001
Respiratory	1196 (0.9)	102 (1.1)	0.159
Thrombotic	1600 (1.3)	125 (1.4)	0.310
Renal	6950 (5.5)	478 (5.3)	0.463
30‐day complication (any)			
Any major complication	41,340 (32.4)	2853 (31.4)	0.057
MI	13,667 (10.7)	791 (8.7)	< 0.001
Respiratory	1614 (1.3)	145 (1.6)	0.007
Thrombotic	3242 (2.5)	260 (2.9)	0.059
Renal	8102 (6.4)	565 (6.2)	0.636
90‐day complication (any)			
Any major complication	44,988 (35.3)	3150 (34.7)	0.285
MI	14,314 (11.2)	847 (9.3)	< 0.001
Respiratory	1989 (1.6)	195 (2.2)	< 0.001
Thrombotic	4676 (3.7)	381 (4.2)	0.009
Renal	9214 (7.2)	643 (7.1)	0.625
Readmission (30‐day)	12,240 (9.6)	1012 (7.6)	< 0.001
Readmission (90‐day)	17,552 (13.7)	1410 (7.4)	< 0.001
LOS (day)	6.73 [6.70–6.77]	6.72 [6.57–6.87]	0.839
Pre‐surgery costs ($)	12,814.8 [12,713.9–12,915.8]	17,340.7 [16,897.9–17,783.5]	< 0.001
Post‐surgery costs ($)	71,846.9 [71,530.9–72,162.8]	75,915.9 [74,652.9–77,178.9]	< 0.001

*Note:* Outcomes are reported at index hospitalization and 30 days and 90 days after surgery.

Abbreviations: LOS, length of stay; SD, standard deviation; USD, U.S. dollars.

**FIGURE 1:**
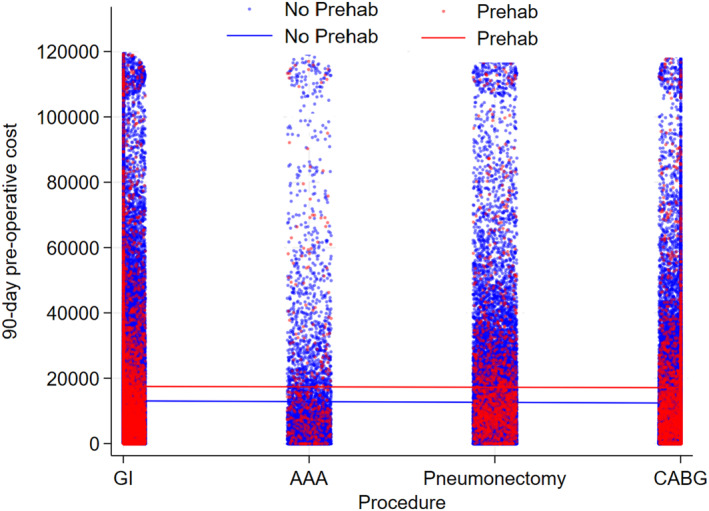
Ninety‐day preoperative cost by procedure and prehabilitation status. Scatter points show patient‐level costs (jittered for visibility); solid lines are linear fits within each group. Blue, no prehabilitation and red, prehabilitation. *Y*‐axis truncated at $120,000 for readability. Procedures: AAA, abdominal aortic aneurysm repair; CABG, coronary artery bypass grafting; GI, gastrointestinal surgery.

**FIGURE 2:**
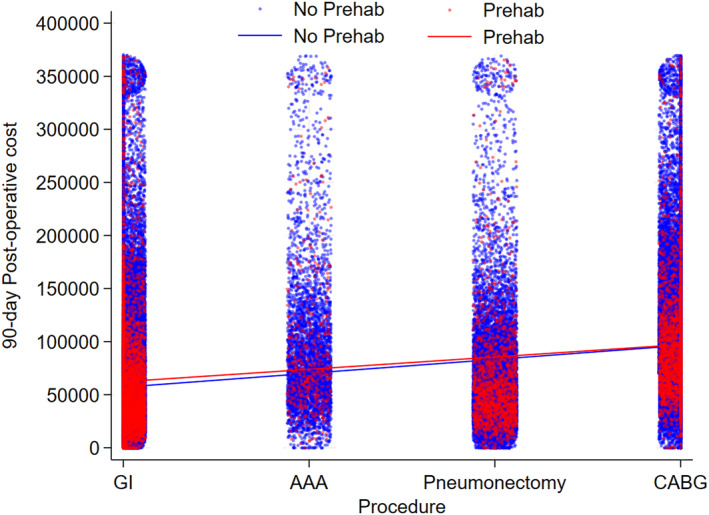
Ninety‐day postoperative cost by procedure and prehabilitation status. Scatter points show patient‐level costs (jittered); solid lines are linear fits within each group. Blue, no prehabilitation; red, prehabilitation. *Y*‐axis scaled to $400,000. Procedures: AAA, abdominal aortic aneurysm repair; CABG, coronary artery bypass grafting; GI, gastrointestinal surgery.

Patients who had received at least one prehabilitation service were also less often readmitted within 30 (7.6% vs. 9.6%) and 90 (7.6% vs. 13.8%) days (both *p* < 0.001). Despite this, median costs of the overall episode of care were higher among patients receiving prehabilitation during both the preoperative ($17,341 [IQR 16,898–17,784] vs. $12,815 [IQR 12,714–12,916]) and postoperative ($75,915 [IQR 74,653–77,179] vs. no $71,847 [IQR 71,531–72,163]) periods (both *p* < 0.001) (Figures 1 and 2).

### 3.3 Adjusted Association Between Prehabilitation and Outcomes

On multivariable analyses, prehabilitation was associated with lower odds of any complication during the index hospitalization (aOR 0.94, 95% CI 0.90–0.99), while no differences were observed at 30‐ or 90‐days (*p* > 0.05) (Table 3). Patients who received prehabilitation had 22% lower odds of MI during the index hospitalization (aOR 0.78, 95% CI 0.71–0.85), as well as 21% and 19% lower likelihood at 30 (aOR 0.79, 95% CI 0.72–0.86) and 90 (aOR 0.81, 95% CI 0.74–0.89) days, respectively. During the 90‐day postoperative window, prehabilitation patients had higher odds of respiratory (aOR 1.29, 95% CI 1.11–1.49) and thromboembolic complications (aOR 1.13, 95% CI 1.02–1.26). Prehabilitation was associated with higher odds of 30‐day readmission (aOR 1.18, 95% CI 1.10–1.26) and 90‐day readmission (aOR 1.15, 95% CI 1.08–1.22). Prehabilitation was, however, associated with higher adjusted costs both in the preoperative ($4227, 95% CI $3834–$4621) and postoperative ($4019, 95% CI $2866–$5173) periods.

**TABLE 3:** Adjusted associations between prehabilitation and postoperative outcomes.

Outcomes (ref: No prehabilitation)	AOR/*β* (95% CI)	*p*‐value
Index date complication (any)		
Any major complication	0.94 (0.90–0.99)	0.047
MI	0.78 (0.71–0.85)	< 0.001
Respiratory	1.12 (0.91–1.37)	0.914
Thrombotic	1.08 (0.90–1.30)	0.383
Renal	0.99 (0.89–1.09)	0.848
30‐day complication (any)		
Any major complication	0.98 (0.94–1.03)	0.610
MI	0.79 (0.72–0.86)	< 0.001
Respiratory	1.17 (0.99–1.39)	0.065
Thrombotic	1.11 (0.98–1.27)	0.086
Renal	1.00 (0.91–1.09)	0.905
90‐day complication (any)		
Any major complication	1.00 (0.95–1.05)	0.824
MI	0.81 (0.74–0.89)	< 0.001
Respiratory	1.29 (1.11–1.49)	0.001
Thrombotic	1.13 (1.02–1.26)	0.017
Renal	1.00 (0.92–1.08)	0.980
Readmission (30 days)	1.18 (1.10–1.26)	< 0.001
Readmission (90‐day)	1.15 (1.08–1.22)	< 0.001
LOS (day)	0.11 (−0.02 to 0.25)	0.111
Pre‐surgery costs ($)	4227.4 [3833.7–4621.0]	< 0.001
Post‐surgery costs ($)	4019.6 [2865.9–5173.3]	< 0.001

*Note:* OR with 95% CIs are reported. Continuous outcomes (LOS, costs) were modeled using generalized linear models.

Abbreviations: aOR, adjusted odds ratio; CI, confidence interval; LOS, length of stay; USD, U.S. dollars.

On stratified analyses, a slight dose–effect relationship was observed related to number of prehabilitation modalities, as two‐modality prehabilitation was associated with lower odds of any major complication during the index hospitalization (aOR 0.90, 95% CI 0.84–0.96) than no prehabilitation, as well as with reduced odds of MI at the index (aOR 0.77, 95% CI 0.69–0.87) and 30‐day (aOR 0.79, 95% CI 0.70–0.88), and 90‐day (aOR 0.81, 95% CI 0.72–0.90) intervals. Similar reductions in MI were observed among patients receiving ≥ 3 prehabilitation modalities (Table 4).

**TABLE 4:** Stratified analysis of postoperative outcomes by prehabilitation modality (number of prehabilitation modalities).

Outcomes (ref: No prehabilitation)	AOR (95% CI)	*p*‐value	AOR (95% CI)	*p*‐value
	2 prehabilitation modalities		> 2 prehabilitation modalities	
Index date complication (any)				
Any major complication	0.90 (0.84–0.96)	0.003	1.02 (0.94–1.10)	0.618
MI	0.77 (0.69–0.87)	< 0.001	0.78 (0.68–0.90)	0.001
Respiratory	1.02 (0.78–1.34)	0.828	1.27 (0.93–1.72)	0.121
Thrombotic	0.95 (0.74–1.21)	0.704	1.29 (0.99–1.68)	0.058
Renal	0.99 (0.87–1.12)	0.906	0.98 (0.85–1.14)	0.871
30‐day complication (any)				
Any major complication	0.95 (0.89–1.01)	0.121	1.04 (0.96–1.12)	0.272
MI	0.79 (0.70–0.88)	< 0.001	0.79 (0.69–0.91)	0.001
Respiratory	1.11 (0.89–1.38)	0.346	1.28 (0.98–1.66)	0.063
Thrombotic	0.98 (0.83–1.17)	0.907	1.32 (1.09–1.58)	0.003
Renal	1.01 (0.90–1.13)	0.830	0.99 (0.86–1.14)	0.941
90‐day complication (any)				
Any major complication	0.97 (0.91–1.03)	0.388	1.05 (0.98–1.13)	0.151
MI	0.81 (0.72–0.90)	< 0.001	0.82 (0.72–0.94)	0.005
Respiratory	1.26 (1.04–1.52)	0.016	1.33 (1.06–1.68)	0.014
Thrombotic	1.07 (0.93–1.23)	0.293	1.23 (1.05–1.44)	0.010
Renal	1.02 (0.91–1.13)	0.697	0.97 (0.85–1.10)	0.658
LOS (day)	0.025 [−0.14 to 0.19]	0.26	0.24 [0.02–0.45]	0.26
Pre‐surgery costs ($)	3586.6 [3086.5–4086.6]	< 0.001	5199 [4588.0–5810.7]	< 0.001
Post‐surgery costs ($)	3458.7 [1993.1–4924.3]	< 0.001	4870.4 [3078.6–6662.2]	< 0.001

*Note:* ORs with 95% CIs are reported for binary outcomes. Continuous outcomes were modeled with generalized linear models.

Abbreviations: CI, confidence interval; LOS, length of stay; OR, odds ratio; USD, U.S. dollars.

When stratified by surgical procedure, prehabilitation prior to CABG was associated with the lowest odds of complications at index hospitalization (aOR 0.83, 95% CI 0.76–0.90) and 30 (aOR 0.85, 95% CI 0.78–0.93) and 90 (aOR 0.89, 95% CI 0.82–0.97) days (Table 5).

**TABLE 5:** Procedure‐specific adjusted associations between prehabilitation and outcomes.

Outcomes (ref: No prehabilitation)	aOR (95% CI)	*p*‐value	aOR (95% CI)	*p*‐value	aOR (95% CI)	*p*‐value	AOR (95% CI)	*p*‐value
	GI surgeries		AAA		Pneumonectomy		CABG	
Index date complication (any)								
Any major complication	1.07 (0.99–1.15)	0.07	1.00 (0.77–1.30)	0.963	0.91 (0.79–1.06)	0.242	0.83 (0.76–0.90)	< 0.001
MI	1.2 (0.72–2.02)	0.452	0.87 (0.34–2.19)	0.771	0.72 (0.29–1.79)	0.484	0.77 (0.70–0.84)	< 0.001
Respiratory	1.44 (1.01–2.04)	0.039	1.07 (0.45–2.51)	0.873	1.01 (0.68–1.48)	0.954	1.03 (0.72–1.48)	0.857
Thrombotic	1.11 (0.86–1.42)	0.400	1.43 (0.70–2.930	0.319	1.08 (0.70–1.67)	0.720	1.00 (0.67–1.49)	0.990
Renal	1.01 (0.87–1.18)	0.826	0.94 (0.64–1.39)	0.793	1.13 (0.82–1.55)	0.447	0.95 (0.82–1.10)	0.549
30‐day complication (any)								
Any major complication	1.09 (1.01–1.16)	0.013	1.15 (0.91–1.44)	0.226	0.96 (0.84–1.10)	0.613	0.85 (0.78–0.925)	< 0.001
MI	1.14 (0.73–1.78)	0.557	1.05 (0.52–2.12)	0.881	0.68 (0.29–1.56)	0.366	0.78 (0.71–0.85)	< 0.001
Respiratory	1.44 (1.06–1.96)	0.018	0.91 (0.41–2.01)	0.832	1.11 (0.80–1.52)	0.519	1.13 (0.84–1.52)	0.395
Thrombotic	1.13 (0.94–1.35)	0.180	1.58 (0.96–2.61)	0.070	1.06 (0.77–1.47)	0.698	1.07 (0.83–1.38)	0.572
Renal	1.04 (0.91–1.19)	0.524	0.95 (0.67–1.36)	0.815	1.11 (0.82–1.50)	0.466	0.96 (0.83–1.10)	0.588
90‐day complication (any)								
Any major complication	1.07 (1.01–1.15)	0.023	1.10 (0.88–1.37)	0.397	1.00 (0.88–1.14)	0.922	0.89 (0.82–0.97)	< 0.001
MI	1.21 (0.72–2.02)	0.452	0.87 (0.34–2.19)	0.771	0.72 (0.29–1.79)	0.484	0.77 (0.70–0.84)	< 0.001
Respiratory	1.69 (1.31–2.18)	< 0.001	0.92 (0.46–1.86)	0.835	1.07 (0.80–1.44)	0.611	1.28 (1.00–1.65)	0.050
Thrombotic	1.05 (0.90–1.22)	0.494	1.45 (0.93–2.27)	0.097	1.15 (0.88–1.49)	0.298	1.28 (1.04–1.57)	0.016
Renal	1.02 (0.90–1.16)	0.659	0.96 (0.68–1.34)	0.818	1.07 90.82–1.40)	0.598	0.97 (0.85–1.10)	0.672
LOS (day)	0.27 (0.07–0.48)	0.007	−0.02 (−0.67 to 0.61)	0.928	−0.04 (−0.39 to 0.31)	0.823	−0.019 (−0.23 to 0.19)	0.855
Pre‐surgery costs ($)	4444.2 [3905.4–4983.0]	< 0.001	3560.1 [1388.7–5731.6]		2613.5 [1457.6–3769.4]	< 0.001	4706.2 [4016.1–5396.2]	< 0.001
Post‐surgery costs ($)	5624.0 [4124.2–7123.8]	< 0.001	−2686.1 [−9549.9 to 4177.5]	0.443	4954.4 [2121.7–7787.2]	0.001	1463.2 [−914.7 to 3841.2]	0.228

*Note:* ORs with 95% CIs are shown for binary outcomes; *β* coefficients with 95% CIs are shown for continuous outcomes.

Abbreviations: AAA, abdominal aortic aneurysm; CABG, coronary artery bypass graft; CI, confidence interval; GI, gastrointestinal; LOS, length of stay; OR, odds ratio; USD, U.S. dollars.

## 4 Discussion

Structured preoperative prehabilitation has been increasingly integrated into ERAS and value‐based care frameworks, raising questions about its real‐world effectiveness across major operations [24]. Prior trials and meta‐analyses suggested reductions in cardiopulmonary complications and modest decreases in LOS with targeted exercise and inspiratory muscle training. However, heterogeneous program content, referral bias, and the predominance of small single‐center studies limit generalizability and leave uncertainty about effects on overall morbidity, readmissions, and costs [1, 11, 14, 25]. A major strength of the current study has been the use of a large national claims database including more than 136,000 patients across multiple high‐risk operations, which has enabled evaluation of prehabilitation in routine practice rather than within a single‐center protocolized setting. In addition, the modality‐stratified, procedure‐specific, and cost analyses have provided a broader assessment of effectiveness and value than has been available in many prior studies. Of note, prehabilitation was associated with several notable benefits, particularly reduced cardiac complications; however, benefit was not uniform across postoperative outcomes, as adjusted readmission and perioperative costs were higher. In addition, the most favorable observed associations were seen among patients undergoing CABG, reducing the incidence of MI the most among all complications examined. These demonstrate that prehabilitation, while beneficial in certain surgical procedures such as CABG, yet may not have as pronounced an effect among individuals undergoing other operations. In essence, while improving certain outcomes (e.g., MI), the effect of prehabilitation was not uniform across all complication types or patient subgroups. These subgroup findings should be interpreted as exploratory and hypothesis‐generating given the observational design and potential for residual confounding.

One consistent benefit of prehabilitation related to cardiovascular risk as recipients of rehabilitation had lower adjusted odds of postoperative MI (Table 3). A core goal of prehabilitation is to enhance cardiopulmonary reserve and enhance patient tolerance to cardiovascular perioperative stress. To this point, patients who received prehabilitation had 22% lower odds of MI during index hospitalization, as well as 21% and 19% lower likelihood at 30 and 90 days, respectively. Of note, the protective effect was most pronounced among patients undergoing CABG without incurring higher episode costs (Table 5). These effects are biologically plausible as exercise‐centered “prehab cardiac rehab” improves aerobic capacity, pulmonary mechanics, and cardiorespiratory reserve during surgical recovery following a cardiac procedure [19]. Interestingly, the benefits of prehabilitation were not as pronounced among patients undergoing noncardiac related surgical procedures. For example, prehabilitation did not reduce composite morbidity following a major gastrointestinal surgical procedure for a malignant indication. These data suggest that recovery may be driven by different mechanisms, including chronic cancer‐related catabolic processes, which may mitigate the effects of any short‐interval preoperative conditioning [19, 26]. In turn, a pragmatic, risk‐aligned strategy is warranted that prioritizes exercise and nutrition as core components, adding respiratory or behavioral elements selectively (e.g., inspiratory training for thoracic procedures, nutritional optimization for GI procedures, and cardiovascular rehab for cardiac operations) [27]. Future work should delineate synergistic modality combinations, identify high‐yield subgroups, and define optimal dose and timing of prehabilitation. Importantly, the association between prehabilitation and postoperative outcomes was not uniform across complication domains, as lower odds of myocardial infarction coexisted with higher odds of 90‐day respiratory and thromboembolic events. The higher odds of 90‐day respiratory and thromboembolic events may have reflected residual confounding by indication, because patients referred for prehabilitation may have had poorer baseline pulmonary reserve, reduced mobility, smoking burden, or other unmeasured risk factors that were not fully captured in claims. In addition, these outcomes were identified from administrative codes over a 90‐day window and may have been influenced by differential post‐discharge surveillance and coding intensity. Accordingly, these findings should not be interpreted as evidence that prehabilitation directly increased the risk of pulmonary or thromboembolic complications.

Although unadjusted readmission rates were lower among patients who received prehabilitation, adjusted analyses demonstrated slightly higher odds of both 30‐ and 90‐day readmission (Table 3). Prior studies have reported lower postoperative utilization after prehabilitation in selected settings, including thoracic surgery; however, the current findings suggest that this benefit may not generalize uniformly across procedures in routine practice [14, 28, 29, 30, 31]. Given the widespread ERAS adoption, delineating the independent effect of prehabilitation apart from contemporaneous perioperative care pathways remains challenging [32, 33]. The reversal between unadjusted and adjusted readmission findings may reflect residual confounding, referral patterns, or unmeasured differences in baseline complexity and post‐discharge care needs among patients selected for prehabilitation who were not fully captured in claims. As such, the current data do not support the conclusion that prehabilitation reduces readmission in routine practice, and the relationship between prehabilitation, care coordination, and post‐discharge utilization warrants further prospective study. Prehabilitation should therefore be viewed as a potential complement to, rather than a substitute for, established ERAS pathways. In addition, perioperative costs were higher among patients who received prehabilitation, likely reflecting program‐related expenditures, greater preoperative healthcare engagement, and possible selection of more complex patients for intervention [25, 34]. Prior studies evaluating the economic impact of prehabilitation have reported heterogeneous findings across settings and follow‐up horizons [25, 35]. From a health system perspective, reductions in myocardial infarction may offset some downstream utilization; however, because costs were measured within a 90‐day perioperative window and no formal cost‐effectiveness analysis was performed, these findings should not be interpreted as demonstrating net long‐term savings. Further work is needed to identify which patients, procedures, and program components are most likely to provide clinical benefit in a cost‐conscious manner.

Findings of the current study should be interpreted in light of certain limitations. Given the retrospective nature of the study, there may have been residual confounding despite adjustment for other clinical factors. Data on race/ethnicity, frailty, baseline function, nutrition, smoking intensity, ERAS adherence, and practice patterns were unavailable. Future studies should evaluate whether access to and benefit from prehabilitation differ across sociodemographic and geographic subgroups, including sex, race/ethnicity, socioeconomic status, and rurality. Exposure misclassification was possible because self‐directed prehabilitation was not billed; claims also lacked detail on intensity, adherence, quality, and precise timing [36]. This source of exposure misclassification would likely have biased observed associations toward the null. Outcomes based on diagnosis/procedure codes may be susceptible to coding error, although MarketScan has been validated in multiple previous surgical outcome studies [37, 38]. Costs reflected payer payments and excluded societal costs, while commercial coverage limited generalizability.

In conclusion, prehabilitation before major surgery was associated with lower odds of index complications, particularly postoperative myocardial infarction, but was not associated with uniform benefit across all complication types and was associated with higher perioperative costs. The most favorable associations were observed among CABG patients, but these procedure‐specific findings should be interpreted cautiously and require prospective confirmation before supporting targeted implementation strategies. Prospective studies should refine patient selection, optimize modality combinations, and evaluate long‐term cost‐effectiveness to guide integration of prehabilitation into routine perioperative care.

## Author Contributions

**Meher Angez:** conceptualization, investigation, writing – original draft, methodology, writing – review and editing, software, formal analysis, supervision, resources, data curation. **Zayed Rashid:** conceptualization, writing – original draft, methodology, writing – review and editing, resources. **Odysseas P. Chatzipanagiotou:** conceptualization, writing – original draft, writing – review and editing, methodology, software, formal analysis. **Areesh Mevawalla:** writing – original draft, conceptualization, methodology, software, writing – review and editing, formal analysis. **Elemosho Abdulaziz:** conceptualization, writing – original draft, writing – review and editing, methodology, software. **Qaidar Alizai:** conceptualization, writing – original draft, methodology, writing – review and editing, software. **Timothy M. Pawlik:** conceptualization, investigation, writing – original draft, writing – review and editing, methodology, software, formal analysis, supervision, resources, data curation, visualization.

## Funding

The authors have nothing to report.

## Conflicts of Interest

The authors declare no conflicts of interest.

## Supporting information


Supporting Information S1

## Data Availability

This study used licensed data from the IBM MarketScan Commercial Database. There are restrictions on the availability of these data, and the data can be accessed with permission from the IBM MarketScan Commercial Database.
