# Supplementary material for: Prehabilitation and Postoperative Outcomes in Major Surgery: A Retrospective Cohort Study Using MarketScan Claims Data
Source: World J Surg. 2026 Jun 9;50(7):1835–45. doi: 10.1002/wjs.70437 (PMC13356519; doi:10.1002/wjs.70437)
Supplement: Supplementary file 1 — Supporting Information S1 [file WJS-50-1835-s001.docx]

**Supplementary Table S1. ICD-10-PCS Root-Code Prefixes Used to Identify Major Surgical Procedures**

| **Procedure** | **ICD-10-PCS root-code prefixes** |
| --- | --- |
| **Coronary artery bypass grafting** | 0210, 0211, 0212, 0213 |
| **Open abdominal aortic aneurysm repair** | 02RF, 0410, 0450, 04B0, 04R0, 04U0, 04V0, X2RF |
| **Pneumonectomy** | 01B3, 01BL, 0B5C, 0B5D, 0B5F, 0B5G, 0B5H, 0B5J, 0B5K, 0B5L, 0B5M, 0BBC, 0BBD, 0BBF, 0BBG, 0BBH, 0BBJ, 0BBK, 0BBL, 0BBM, 0BTC, 0BTD, 0BTF, 0BTG, 0BTH, 0BTJ, 0BTK, 0BTL, 0BTM |
| **Pancreatectomy** | 0FBD, 0FBF, 0FBG, 0FTD, 0FTF, 0FTG |
| **Colectomy** | 0DBE, 0DBF, 0DBG, 0DBH, 0DBK, 0DBL, 0DBM, 0DBN, 0DTE, 0DTF, 0DTG, 0DTH, 0DTK, 0DTL, 0DTM, 0DTN |

**Supplementary Table S2. International Classification of Diseases, Tenth Revision Diagnosis and Procedure Codes Used to Identify Perioperative Complications.**

| **Complication** | **ICD-10 Diagnosis Codes** | **ICD-10 Procedure Codes** |
| --- | --- | --- |
| Pulmonary failure | J96.00, J96.90, J96.20, J80, R06.03 | 0BH17EZ, 0BH18EZ, 0B717DZ, 0B718DZ, 0BH07DZ, 0BH07YZ, 0BH172Z, 0BH17YZ, 0BH182Z, 0BH18YZ, 0BHK7YZ, 0BHK8YZ, 0BHL7YZ, 0BHL8YZ, 0WHQ7YZ, 5A1935Z, 5A1945Z, 5A1955Z |
| Pneumonia | J13, J14, J15.xxx, J16.xx, J17, J18.xx, J69.xx | — |
| Myocardial infarction | I21.xx | — |
| Deep venous thrombosis or pulmonary embolism | I26.xx, I80.xxx | — |
| Acute kidney failure | N17.x | 5A1D70Z, 5A1D80Z, 5A1D90Z |
